# *Drosophila* Heartless Acts with Heartbroken/Dof in Muscle Founder Differentiation

**DOI:** 10.1371/journal.pbio.0030337

**Published:** 2005-09-06

**Authors:** Devkanya Dutta, Sanjeev Shaw, Tariq Maqbool, Hetal Pandya, K VijayRaghavan

**Affiliations:** **1**National Centre for Biological Sciences, Tata Institute of Fundamental Research, Bangalore, India; University of CologneGermany

## Abstract

The formation of a multi-nucleate myofibre is directed, in *Drosophila,* by a founder cell. In the embryo, founders are selected by Notch-mediated lateral inhibition, while during adult myogenesis this mechanism of selection does not appear to operate. We show, in the muscles of the adult abdomen, that the Fibroblast growth factor pathway mediates founder cell choice in a novel manner. We suggest that the developmental patterns of Heartbroken/Dof and Sprouty result in defining the domain and timing of activation of the Fibroblast growth factor receptor Heartless in specific myoblasts, thereby converting them into founder cells. Our results point to a way in which muscle differentiation could be initiated and define a critical developmental function for Heartbroken/Dof in myogenesis.

## Abstract 2

In the fly embryo, the founder cells that direct myofibre formation are selected through Notch-mediated signaling. The authors show that in adult animals, founder cells are specified by signaling through the FGF pathway.

## Introduction

Each multi-nucleate muscle fibre in an animal is uniquely positioned and performs a specific function. The development of these features is a consequence of the specification of the identity of the fibre, and its differentiation in the context of its innervation and attachment to tendon cells. In *Drosophila,* the identity of a muscle fibre is specified by the expression of a combination of transcription factors unique to each muscle and by its location [1–6]. In addition to characteristics that specify the identity of each muscle, all syncytial muscles in flies share a common mechanism of fibre formation. This common mechanism uses a special cell, the founder cell, that organises the fusion process and provides it directionality [1,4,7–9]. A founder cell attracts its neighbouring myoblasts—the “fusion-competent” myoblasts—that fuse with the founder, to form a multi-nucleate myotube. Specific molecules expressed in the founder and fusion-competent cells direct the fusion process [7,10]. One such molecule, expressed on the surface of the founder cells and encoded by the gene *dumbfounded (duf*, also called *kin of irre* or *kirre),* is an Ig domain containing membrane protein that interacts with other Ig domain proteins expressed on the surface of fusion-competent cells [11,12]. This interaction initiates the process of cell fusion. The selection of founder cells and the expression of *duf* in founder cells are thus important first steps in muscle differentiation.

In the embryo, a founder cell is selected from a cluster of equivalent myoblasts that are specified by activation of the Ras/mitogen-activated protein kinase (MAPK) pathway. From this “equivalent group”, a single cell—the precursor cell—is chosen by Notch-mediated lateral inhibition. The precursor cell divides to give rise to two embryonic founder cells or an embryonic founder cell and an adult myoblast progenitor [1,2,4,13]. Founder cells, thus specified, express transcription factors unique to each founder, but all founders express the *duf-lacZ* reporter gene [14]. The remaining cells of the equivalent group constitute the fusion-competent myoblasts.

The muscles of the adult fly are also made using the founder mechanism [15–17]. The formation of each adult muscle fibre appears to be nucleated by a *duf-lacZ*-expressing cell, and these cells exhibit features characteristic of founders [15]. In adult myogenesis, *duf-lacZ* expression in a founder pattern is currently the earliest available molecular marker for the formation of muscle founder. The monoclonal antibody 22C10 has also been shown to be a marker of muscle founder cells in the adult abdomen [15]. The antibody 22C10 recognises the microtubule-binding protein Futsch, which has been found to regulate microtubule architecture in neurons during development [18,19]. Futsch, perhaps, serves a similar function in the developing myofibres and its expression in founder cells could indicate this. The pattern of expression of *duf-lacZ* during myofibre formation and the function of *duf-lacZ*-expressing cells suggest that the founder cell mechanism is conserved through *Drosophila* development and could, perhaps, be even more generally applicable [15].

**Figure 1:**
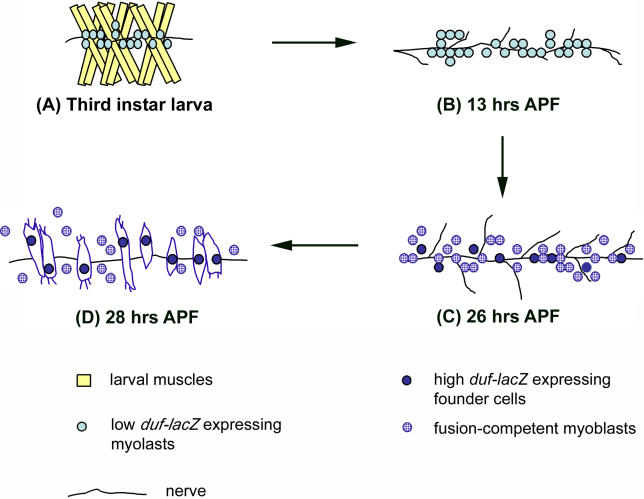
Schematic Representation of the Appearance of Founder Cells in the Abdomen (A) Late third instar larva. Precursor myoblasts for the adult abdominal muscles are associated with the segmental and inter-segmental nerves. These myoblasts express *duf-lacZ* in low levels. (B) 13 h APF. The adult myoblasts, while continuing to express low levels of *duf-lacZ,* proliferate and migrate out along the nerve. (C) 26 h APF. By this stage, low *duf-lacZ* expression in all myoblasts is replaced by higher levels of *duf-lacZ* in selected cells, the founder cells. The remaining cells, termed the fusion-competent cells, eventually fuse with the founder cells to form the adult myotubes. (D) 28 h APF. The nascent myotubes, founded by the founder cells, can be visualised by staining with the antibody 22C10.

Significantly, though, the mechanism of founder cell selection during adult myogenesis is different from that used in the embryo: Notch-mediated lateral inhibition does not appear to be used in adult founder cell choice [15]. The embryonic origin of adult myoblasts [4,20,21]—they are clonally derived from siblings of embryonic founder cells—make this understandable, in hindsight. Indeed, in the third larval instar, adult myoblasts in the abdomen, seen associated with nerves, all express *duf-lacZ* at low levels [15]. During pupal development most myoblasts completely shut down *duf-lacZ* expression while some begin to express this reporter in a distinct founder cell pattern [15]. This is shown schematically in Figure 1. The question, then, in adult myogenesis is not how a founder cell is chosen from a group of apparently naïve myoblasts, but how founder properties are up-regulated in a defined set of myoblasts at the sites of fibre formation, while properties characteristic of fusion-competent cells are up-regulated in other cells.

We show here that the maturation of adult founder cells in the abdomen of the fly is mediated by signalling through the Fibroblast growth factor (FGF) receptor Heartless (Htl). In particular, we demonstrate a novel interplay of components of FGF signalling that results in a precise pattern of founder cells for each multi-fibre array of muscles. This interplay involves the regulation of Sprouty (Sty) (a negative regulator of FGF signalling) and Heartbroken (Hbr) (a positive regulator required for Htl signalling).

## Results

Our previous study had shown that Notch-mediated lateral inhibition does not function in the choice of adult founders [15]. We next investigated whether one of the receptor tyrosine kinase (RTK) family of proteins is involved in selection of adult founders. The family of FGF receptors (FGFRs) constitutes an important member of the RTK super-family, and we examined its role in adult founder cell selection. *Drosophila* has two FGFR-coding genes, *htl* [22] and *breathless* (*btl*) [23]. Htl plays a significant part in the patterning of a variety of mesodermal tissues in the embryo. It is required for cell migration during mesodermal invagination, specification of a subset of somatic muscle precursors, and differentiation of heart and midline glial cells [22,24,25]. In the pupa, *htl* mRNA is found to be expressed in the adult thoracic myoblasts [26]. In this study, we examined the involvement of Htl in adult founder selection. Our results illustrate the participation of the Htl pathway in selection of founder cells in the abdomen

### Htl is Expressed in Adult Abdominal Myoblasts

**Figure 2:**
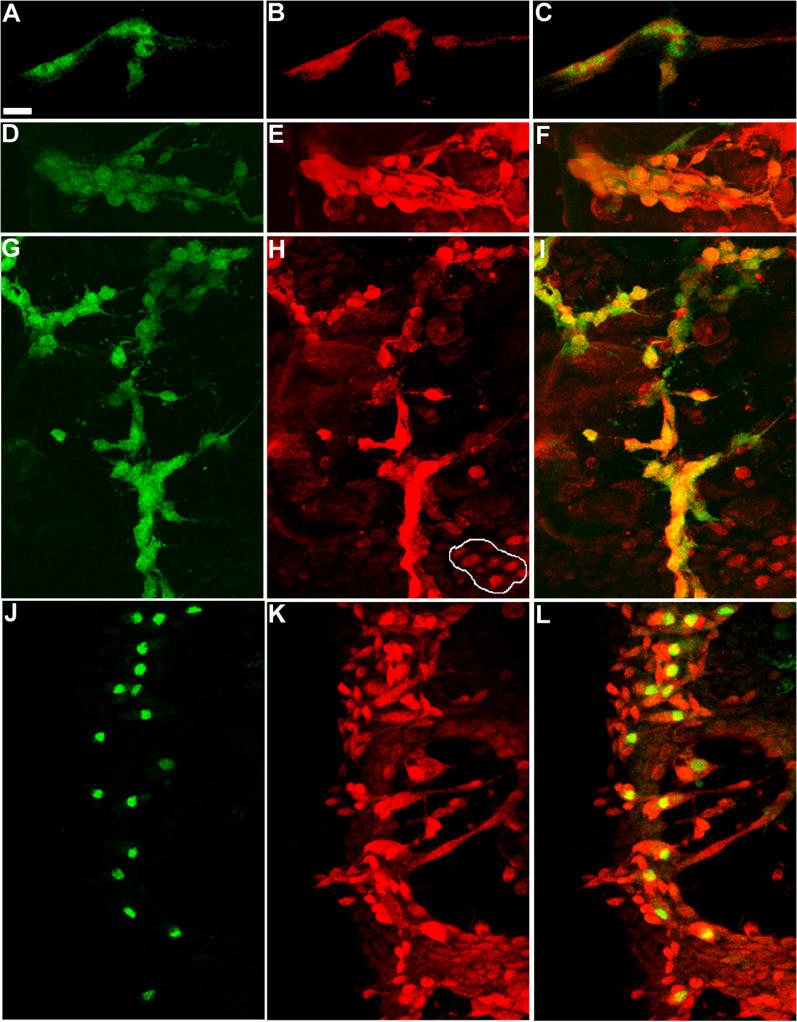
Expression of Htl in Abdominal Mesoderm (A–I) *twi-lacZ* larval or pupal preparations double-labelled with anti-β-galactosidase (green) and anti-Htl (red). (A) *twi-lacZ*-expressing myoblasts in a third instar larva. (B) Same preparation as in (A), showing expression of Htl. (C) Merged image of (A) and (B) showing co-localisation of Htl with *twi-lacZ*. (D–F) A pupal preparation 18 h APF showing a dorsal cluster of *twi*-expressing myoblasts (D) also expressing Htl (E). (F) Merged image of (D) and (E). (G–I) Pupa 18 h APF showing a cluster of lateral myoblasts (G), all expressing Htl (H). (I) Merged image of (G) and (H). Htl expression is also observed in some epidermal cells. A few such cells are outlined in white in (H). (J–L) A *duf-lacZ* pupal preparation 28 h APF double-labelled with anti-β-galactosidase (green) and anti-Htl (red). (J) A set of lateral founders expressing *duf-lacZ*. (K) Htl expression in the same preparation. (L) Merged image of (J) and (K). Htl is present in the founder cells (expressing *duf-lacZ*) and also in the remaining population of fusion-competent myoblasts. Anterior is at top; dorsal midline is at right. Scale Bar = 20 μm.

In the third instar larva, we found that Htl was expressed in the Twist (Twi)–expressing adult abdominal myoblasts that are associated with nerves (Figure 2A–2C). Htl expression in these myoblasts was also observed during pupal development. Figure 2D–2I shows Htl expression at 18 h after puparium formation (APF) in the dorsal (Figure 2D–2F) and lateral (Figure 2G–2I) myoblasts. Double staining with anti-Htl and anti-β-galactosidase antibodies in *duf-lacZ* pupae at 28 h APF showed that Htl continued to be expressed in all myoblasts—in both the *duf-lacZ*-expressing founder cells and in fusion-competent myoblasts (Figure 2J–2L). We also examined the expression of the second *Drosophila* FGFR gene *btl* in the adult mesoderm using a *btl-GAL4* driver. *btl* expression was not observed in abdominal myoblasts or in muscle founder cells (data not shown).

### Modification of Htl Signalling Affects Founder Cell Number in the Abdomen

**Figure 3:**
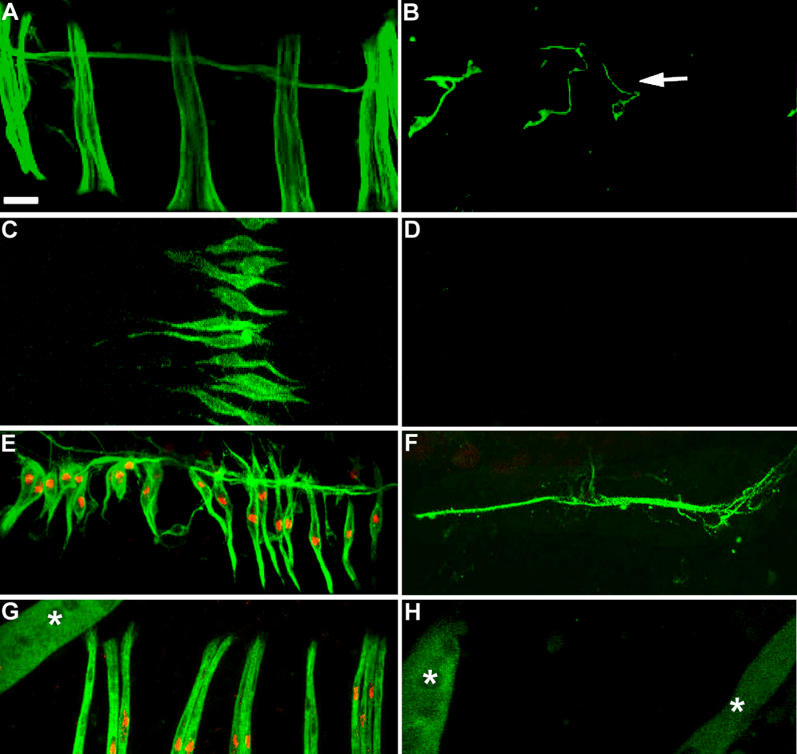
Founder-Pattern upon Decrease in Htl Signaling (A–D) Pupal preparations (grown at 29 °C) stained with 22C10 to visualise the founders. (A) A Canton-S pupa 30 h APF showing a subset of dorsal founders. (B) A similarly aged pupa of the genotype *1151/+*; *UAS-dnhtl/+*; *UAS-dnhtl/+* showing absence of dorsal founders. The white arrow points to a neuronal branching labelled by 22C10. (C) A preparation 28 h APF of Canton-S pupa showing lateral founders. (D) A *1151/+*; *UAS-dnhtl/+*; *UAS-dnhtl/+* pupa, similarly aged as in (C), showing no founders in the lateral hemi-segment. (E and F) A preparation 28 h APF (grown at 29 °C) of a *duf-lacZ* pupa (E) and a *1151/duf-lacZ; UAS-Yan^act^/+* pupa (F) double-labelled with anti-β-galactosidase (red) and 22C10 (green). (E) shows wild-type pattern of a subset of dorsal founders. (F) shows complete absence of founder cells. Only the nerve, stained by 22C10, is visible. (G and H) A preparation 42 h APF (grown at 29 °C) of a *duf-lacZ* pupa (G) and a *1151/duf-lacZ; UAS-Yan^act^/+* pupa (H) double-labelled with anti-β-galactosidase (red) and anti-MHC (green). In contrast to the wild-type pattern of dorsal muscles (G), no muscles are formed in the *UAS-Yan^act^* mis-expression pupa (H). The absence of muscles is not an experimental artefact because in the same preparation the unhistolysed larval muscles [66] can be viewed (white asterisks in [G] and [H]). Anterior is at top; dorsal midline is at left. Scale bar = 20 μm.

All available mutants of Htl are embryonic lethal, making scoring of Htl mutants for pupal or adult phenotypes difficult. We mis-expressed a dominant-negative construct of Htl (dnHtl) to examine the Htl loss-of-function effect in the adult myoblasts (Figure 3). Over-expression of *UAS-dnhtl* using *1151-GAL4,* a GAL4 driver expressed in all adult myoblasts [27,28], resulted in a distinct decrease in the number of founders per hemi-segment. An average of four dorsal founders per hemi-segment, as opposed to wild-type numbers between 17 and 22, were observed in 49% of the hemi-segments, while dorsal founders were completely absent in 17% of these hemi-segments (*n =* 30). Lack of a complete suppression of founder cells in experimental pupae was most likely due to the incomplete penetrance of the dnHtl construct (also reported in [29]). Figure 3B and 3D show dorsal and lateral hemi-segments, respectively, of dnHtl mis-expressed pupae where the founder cells are completely lost. Htl function was additionally compromised by mis-expressing *UAS-htl-RNAi* using the *1151-GAL4* driver. In this case too, a decrease in founder number, similar to that in *dnhtl* mis-expression, was observed (data not shown). Yan, a downstream target of MAPK, functions as an RTK-pathway antagonist. It is an Ets-domain-containing transcription factor that keeps the RTK-responsive genes repressed. Phosphorylation of Yan by MAPK inactivates its function as a repressor [30–33]. We over-expressed an activated form of Yan (Yan^act^), which cannot undergo phosphorylation by MAPK [30], in adult myoblasts using the *1151-GAL4* driver. Yan^act^ mis-expression resulted in a severe phenotype, with no founders being selected (Figure 3F) and no muscle fibres being formed (Figure 3H) in any hemi-segment. Figure 3E and 3G show the respective wild-type patterns.

**Figure 4:**
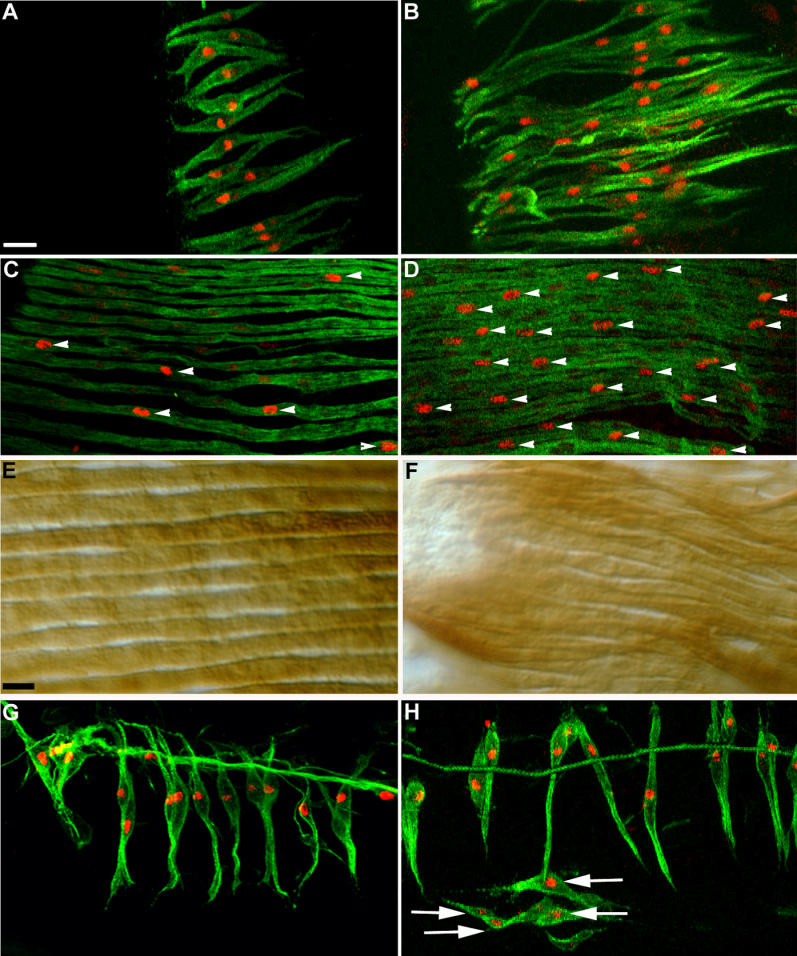
Increase in Founder and Fibre Number upon Activation of Htl Signalling in Myoblasts (A and B) Pupae 28 h APF (grown at 29 °C) double-labelled with anti-β-galactosidase (red) and 22C10 (green). (A) *duf-lacZ* pupa showing wild-type pattern of founders in a section of lateral hemi-segment. The number of founders seen in this section is 13. (B) *1151/duf-lacZ; UAS-*λ*htl/+* pupa showing excess number of lateral founders (26) in an equivalent region. (C and D) Pupae 42 h APF (at 29 °C) double-labelled with anti-β-galactosidase (red) and anti-MHC (green). (C) *duf-lacZ* pupa showing the wild-type lateral myofibres, each having one high *duf-lacZ*-expressing nucleus (white arrowheads). (D) *1151/duf-lacZ; UAS-*λ*htl/+* pupa showing extra number of lateral fibres. White arrowheads indicate high *duf-lacZ*-expressing nuclei within these fibres. For many fibres, in both (C) and (D), the high *duf-lacZ*-expressing nucleus is not within the field of view. (E and F) DIC images of Canton-S (E) and *1151/+*; *UAS-*λ*htl/+* pupae labelled with anti-MHC at 42 h APF. In contrast to the neat array of wild-type muscle fibres (E), myofibres in *UAS-*λ*htl* mis-expressed pupa are heaped in bundles (F). (G) Normal pattern of dorsal founders in *duf-lacZ* pupa at 28 hAPF (at 29 °C). (H) Founder pattern in the dorsal hemi-segment of a *1151/duf-lacZ; UAS-*λ*htl/+* pupa similarly aged to that in (G). White arrows indicate the extra founder cells that have appeared below the normal set of fibres. Anterior is at top; dorsal midline is at left. Scale bar = 20 μm (A–D, G, and H), 13 μm (E and F).

In a converse set of experiments we amplified Htl-mediated signal in myoblasts and obtained a diametrically opposite phenotype, i.e., an increase in number of founders. Over-expression of an activated form of Htl (*UAS-*λ*htl*), using the same *1151-GAL4* driver, led to an increased number of founders and, consequentially, an increased number of muscle fibres in the abdomen (Figure 4). The effect was most prominent for founders for the lateral muscles, where a 2-fold increase in number of founder cells per hemi-segment was observed. Figure 4B shows the excess lateral founders (26 in number) at 28 h APF compared to the control in Figure 4A (13 founders). The excess founder cells were longer and more stretched-out than the normal founders (see Discussion). Figure 4D and 4F show excessive lateral myofibres at 41 h APF (Figure 4C and 4E show the corresponding control preparations). In the dorsal muscles, the excess-founder phenotype was less prominent. Figure 4H shows a region of a dorsal hemi-segment in an activated-Htl pupa. The extra *duf-lacZ*-expressing dorsal founders (white arrows) are observed below the rest of the founders.

Pointed (Pnt), an Ets family protein and a target of MAPK, functions as a positive regulator of the RTK pathway. Upon phosphorylation by MAPK, Pnt outcompetes Yan and turns on genes formerly repressed by Yan [32–34]. Over-expression of an active form of Pnt, therefore, should have the same effect as that of activated Htl. We found that mis-expression of PntP1, an isoform of Pnt [35,36], in adult myoblasts led to an increased number of founder cells compared to the wild-type number in the abdomen (data not shown).

In addition to the FGFRs, another member of the RTK family that is repeatedly used during development to direct cell fate choices is the *Drosophila* Epidermal growth factor receptor (DER) (reviewed in [37]). During embryonic myogenesis, DER provides inductive signals to specify equivalence group for muscle founder cell selection. In the absence of the function of DER or Spitz (a ligand for DER), a large subset of myofibres and their progenitors fail to form in the embryo [13,38] Since both DER and Htl function via the Ras pathway, we wanted to ascertain that the phenotypes observed for the adult founders were specific for the Htl pathway. We decreased DER signalling by mis-expressing a dominant-negative construct of DER (*UAS-dnDER*) using *1151*. Separately, we also over-expressed the DER-inactivating ligand Argos (Aos) [39] in all myoblasts by driving *UAS-aos* using *1151-GAL4*. In both cases, mis-expressed progeny had no abnormality in founder cell number and pattern when compared to wild-type (data not shown).

The above results ruled out the role of DER and confirmed the involvement of the Htl receptor in muscle founder cell choice. However, RTK pathways are also known to function in cell proliferation [40–43]. What, if any, is the contribution of such a role of Htl signalling to the phenotypes we see? The expectation if Htl is not involved in myoblast proliferation is that decreasing Htl signalling in myoblasts should not affect the number of fusion-competent myoblasts. However, since founders are selected from the Twi-expressing myoblast pool, absence of founder cell selection should correspondingly correlate with an increase in myoblast number. We examined the number of Twi-expressing myoblasts at 24 h APF in wild-type pupae and in pupae in which the Htl pathway was down-regulated (Figure S1). In these pupae, the number of myoblasts per hemi-segment did not show a significant change (lateral myoblasts [*n =* 5], wild-type, 58 ± 5, dnHtl, 60 ± 14; dorsal myoblasts [*n =* 4], wild-type, 96 ± 8, dnHtl, 85 ± 11). This ruled out the involvement of the Htl pathway in myoblast proliferation. However, because of the variation in number of myoblasts in control and in experimental pupae, it was not feasible to examine the conversion of specific founders into fusion-competent myoblasts. Activating the pathway using an activated-Htl construct caused a very slight increase in myoblast number (Figure S1) (lateral myoblasts [*n =* 5], wild-type, 58 ± 5, activated Htl, 68 ± 9; dorsal myoblasts [*n =* 4], wild-type, 96 ± 8, activated Htl, 122 ± 11). This effect is, however, still consistent with the Htl pathway not being involved in myoblast proliferation as activated Htl can activate downstream effectors common to an RTK pathway that controls proliferation. In order to examine the effect of GAL4/UAS-induced Htl activation specifically at the time of founder selection, we used a temperature-sensitive GAL80 (TARGET) system [44]. In this system, the activator function of GAL4 protein can be temporally regulated by a ubiquitously expressed temperature-sensitive GAL80 protein (GAL80ts) (functional at 18 °C, non-functional at 29 °C). When animals carrying the *1151-GAL4* driver, a UAS construct for the Htl pathway (either *UAS-dnhtl,* or *UAS-*λ*htl*), and a *tubulin (tub)–GAL80ts* gene were grown at 18 °C, we saw no effect on number of founders or fusion-competent myoblasts (Figure S2). This illustrated that the *tub-GAL80ts* was effective in preventing the function of GAL4 in the pupal mesoderm. The use of the GAL80ts conditional system thus allowed the Htl pathway to be modulated in a controlled background starting with a similar number of myoblasts. *1151-GAL4; tub-GAL80ts; UAS-dnhtl* pupae, raised at 18 °C for 30 h followed by a heat shock at 29 °C for 11 h, were scored for founder and myoblast number. Down-regulation of Htl resulted in no founders being selected (Figure S2). However, the number of fusion-competent myoblasts in each hemi-segment did not show any significant increase (Figure S2) over that in the control (lateral myoblasts, control 54 ± 7, *GAL4-GAL80ts-dnhtl* 62 ± 8; *n =* 4). *1151-GAL4; tub-GAL80ts; UAS-*λ*htl* pupae were grown similarly as above. The expectation was that the activation of Htl would result in an excess of founder cells at the expense of Twi-expressing cells. Indeed, when GAL80 was rendered inactive and the GAL4 used to activate Htl, an excess number of founders were seen (Figure S2). However, myoblast number was comparable to the control (lateral myoblasts in a hemi-segment, control 54 ± 7, *GAL4-GAL80ts–activated Htl* 62 ± 5; *n =* 4), though the pattern of the spatial location of myoblasts was found to be altered (Figure S2). Taken together, we can safely say that the effect of Htl signalling on founder number is a consequence of a direct involvement of the pathway in founder selection and not due to an indirect effect on myoblast number. However, given the large variation in control myoblast numbers (such that conversion of founder cells into fusion-competent myoblasts is not effectively reflected as an increase in myoblasts in dnHtl pupa), and given that not all myoblasts are converted to founders upon activating Htl, it is not feasible to deduce a precise and predictable relationship between numbers of myoblasts and founders.

### Hbr/Dof, a Signalling Protein Specific for *Drosophila* FGFR-Mediated Pathway, Is Expressed in Founder Cells

The continued expression of Htl in adult myoblasts throughout pupal development suggested that restricted presence of receptor protein is not the mechanism behind selection of founder cells from the myoblast pool. We next examined the expression of the cytoplasmic protein Dof [45], also known as Hbr [46] or Stumps [47]. Unlike other intracellular signalling components common to the different RTK-mediated pathways, Hbr/Dof functions exclusively in the FGFR pathway and is essential for the activation of MAPK by the FGFRs [45,46].

**Figure 5:**
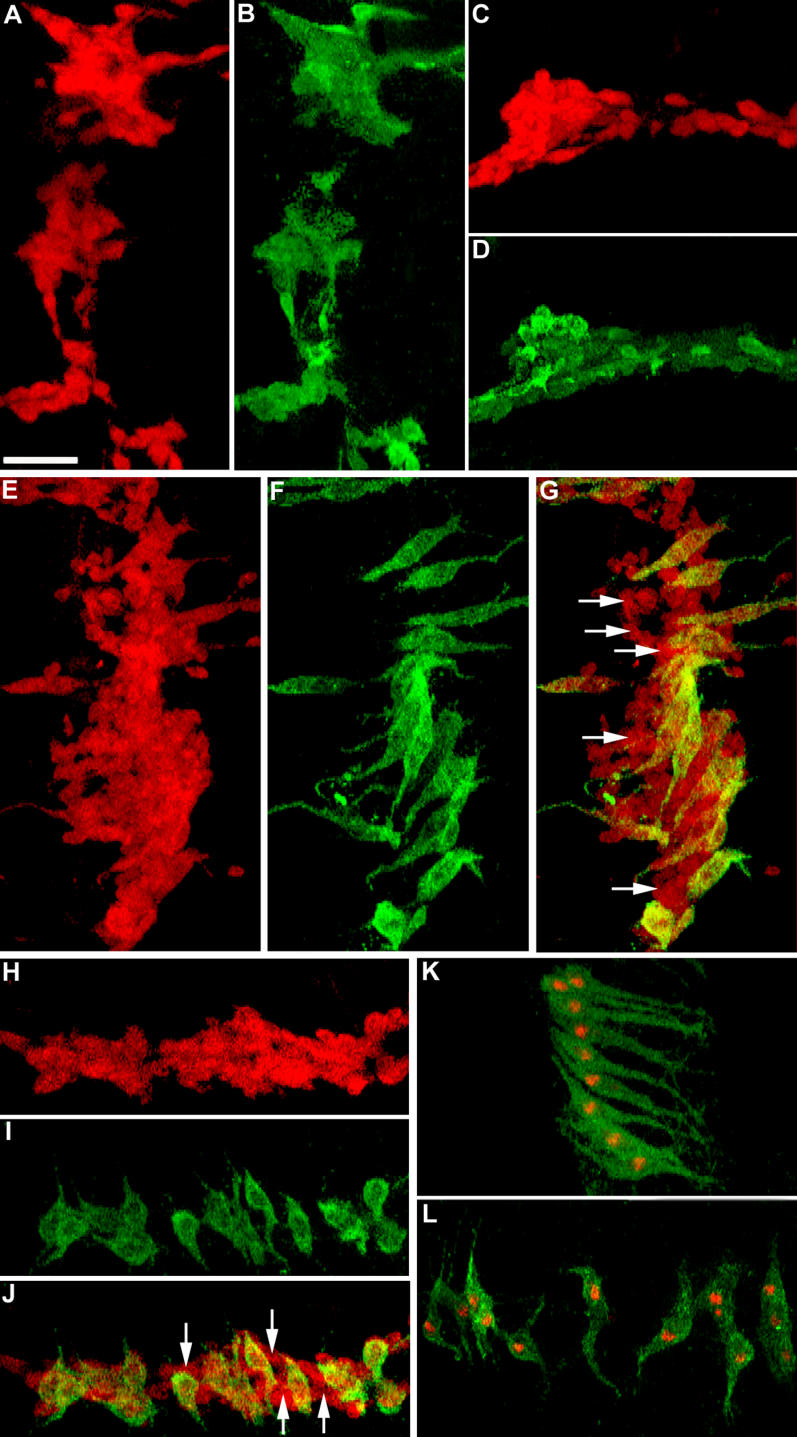
Dof Expression during Abdominal Myogenesis (A–D) *twi-lacZ* pupae 18 h APF double-labelled with anti-β-galactosidase (red) and anti-Dof (green). (A) A cluster of lateral myoblasts. (B) Dof expression in the same lateral cluster, showing expression in all myoblasts. (C) A cluster of dorsal myoblasts. (D) Dof expression in the dorsal cluster. Like in (B), Dof in (D) is expressed in all dorsal myoblasts at this stage. (E–J) *twi-lacZ* pupae 28 h APF double-labelled with anti-β-galactosidase (red) and anti-Dof (green). (E) A cluster of *twi-lacZ*-expressing lateral myoblasts. (F) Dof expression in the same region. (G) Merged image of (E) and (F), showing Dof expression in only a subset of cells. (H) A cluster of *twi-lacZ*-expressing dorsal myoblasts. (I) Dof expression in the same region. (J) Merged image of (H) and (I), showing Dof expression only in selected cells within the cluster. In (G) and (J), the white arrows indicate a few myoblasts that do not express Dof. (K and L) *duf-lacZ* pupae 28 h APF double-labelled with anti-β-galactosidase (red) and anti-Dof (green). The *duf-lacZ*-expressing lateral (K) and dorsal (L) founders express Dof. Thus, the cells that express Dof at 28 h APF are the founder cells. Anterior is at top; dorsal midline is at right. Scale bar = 30 μm.

From the late third instar larva till 24 h APF, Hbr/Dof was found to be expressed in all nerve-associated myoblasts. Figure 5A–5D show expression of Hbr/Dof in myoblasts at 18 h APF; starting 24 h APF, Hbr/Dof expression began to disappear from most myoblasts. By 28 h APF, Hbr/Dof expression was observed in only an array of cells, the founder cells. Figure 5E–5J show Hbr/Dof expression in a restricted number of *twi-lacZ*-expressing cells at 28 h APF while Figure 5K and 5L show Hbr/Dof expression in *duf-lacZ*-expressing founder cells at the same stage (i.e., 28 h APF).

Thus, the pattern of appearance of Hbr/Dof-expressing cells was synchronous with that of the appearance of founder cells. This led us to examine whether Hbr/Dof has a role in imparting founder identity to specific cells.

### Mis-Expression of Hbr/Dof in All Myoblasts Leads to Increased Number of Founders

**Figure 6:**
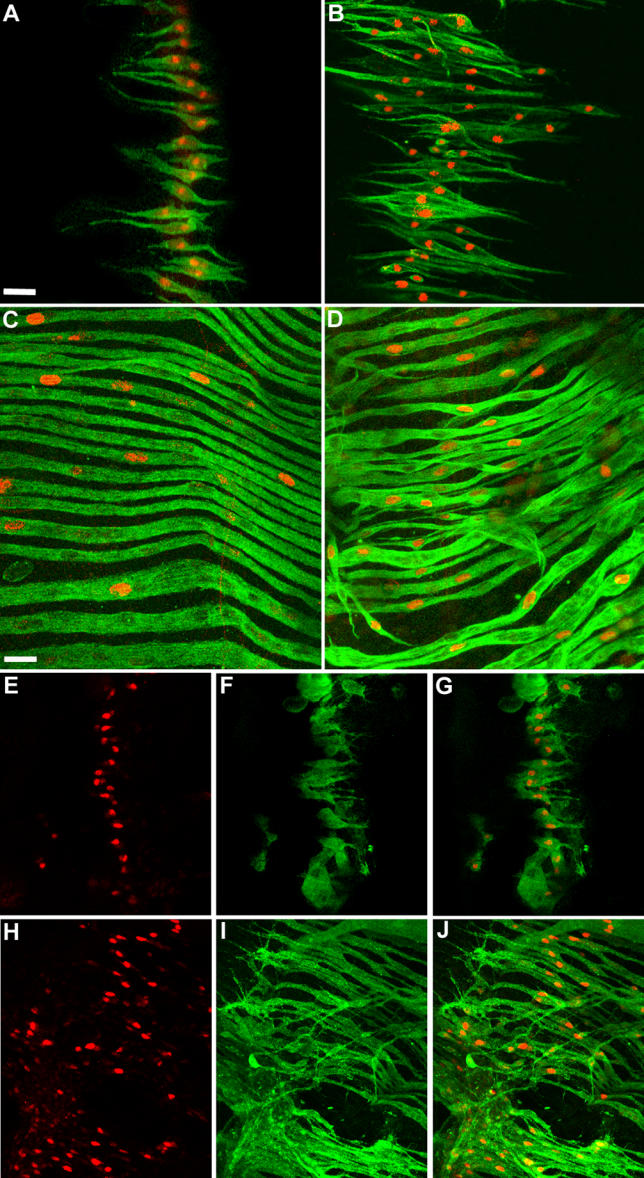
Over-Expression of Hbr/Dof and Htl (A–D) Increase in founder and fibre number upon over-expression of Hbr/Dof in myoblasts. (A and B) Pupae 28 h APF (grown at 29 °C) double-labelled with anti-β-galactosidase (red) and 22C10 (green). (A) *duf-lacZ* pupa showing wild-type pattern of founders in a lateral hemi-segment. There are 19 founders present in the field of view. (B) *1151/duf-lacZ; UAS-dof/+* pupa. Compared to the wild-type, an excess number of founder cells (∼42) are present in the mis-expression pupa. (C and D) Pupae 42 h APF (grown at 29 °C) double-labelled with anti-β-galactosidase (red) and anti-MHC (green). (C) *duf-lacZ* pupa showing lateral muscles in a hemi-segment. (D) *1151/duf-lacZ; UAS-dof/+* pupa showing excessive fibre formation. (E–J) Expression of Dof levels upon activation of Htl. Preparations of *duf-lacZ* (E–G) and *1151/duf-lacZ; UAS-*λ*htl/+* (H–J) pupae double-labelled with anti-β-galactosidase (red) and anti-Dof (green) at 28 h APF (grown at 29 °C). For both pupae, images have been acquired at identical conditions. (E) Lateral founders in one hemi-segment of a *duf-lacZ* pupa. (F) Dof expression in the lateral founders. (G) Merged image of (E) and (F). (H) Lateral founders in an *1151/duf-lacZ; UAS-*λ*htl/+* pupa, showing an increase in founder number. (I) Dof expression in the same pupa. Dof is expressed in the extra founder cells. Also, level of Dof protein in the founders is higher than in the wild-type founders (in F). (J) Merged image of (H) and (I). Anterior is at top; dorsal midline is at right. Scale = 30 μm (A, B, and E–J), 20 μm (C and D).

We maintained expression of Hbr/Dof in all myoblasts by crossing *UAS-dof* with *1151-GAL4*. Hbr/Dof mis-expression led to a dramatic increase in the number of founder cells and muscle fibres in the abdomen compared to the wild-type (Figure 6). Figure 6B is a lateral hemi-segment of an *1151/duf-lacZ; UAS-dof/+* pupa, showing a 2-fold increase in number of founder cells (42) compared to that in a wild-type *duf-lacZ* pupa shown in Figure 6A (19). The excess founders were more elongated in shape than the wild-type founders, similar to the shape observed in activated-Htl pupae (see Figure 4B). Figure 6D shows a *UAS-dof* mis-expressed pupa at 42 h APF having an excess number of lateral myofibres compared to the number in an equivalent region in a similarly aged wild-type pupa (Figure 6C). There are approximately 23 high *duf-lacZ-*expressing nuclei within the field of view in Figure 6D, in contrast to the nine in the wild-type control in Figure 6C. These mis-expression results suggested that restricted expression of Hbr/Dof in pupal abdomen, beginning 24 h APF, is important for specification of adult founders.

### Hbr/Dof Expression Persists in *UAS-*λ*htl* Pupae

We next asked whether the restricted expression of Hbr/Dof is generated by a feedback relationship involving Htl signalling. Such a feedback mechanism has been observed in the embryo where the Ras pathway up-regulates the expression of Hbr/Dof in the muscle progenitor cells [48]. We over-expressed the activated-Htl construct in adult myoblasts (using the *1151-GAL4* driver) and examined the pattern and levels of expression of the Hbr/Dof protein. Over-expression of *UAS-*λ*htl* resulted in a continued expression of Dof in cells, at a level higher than normal. Figure 6E–6G show a 28 h APF *duf-lacZ* pupa expressing Hbr/Dof in founder cells. In a similarly staged *1151*/*duf-lacZ; UAS-*λ*htl/+* pupa, Dof expression was observed in the extra founder cells (Figure 6H–6J).

### *sty,* Initially Present in All Myoblasts, Disappears at the Founder Cell Stage

**Figure 7:**
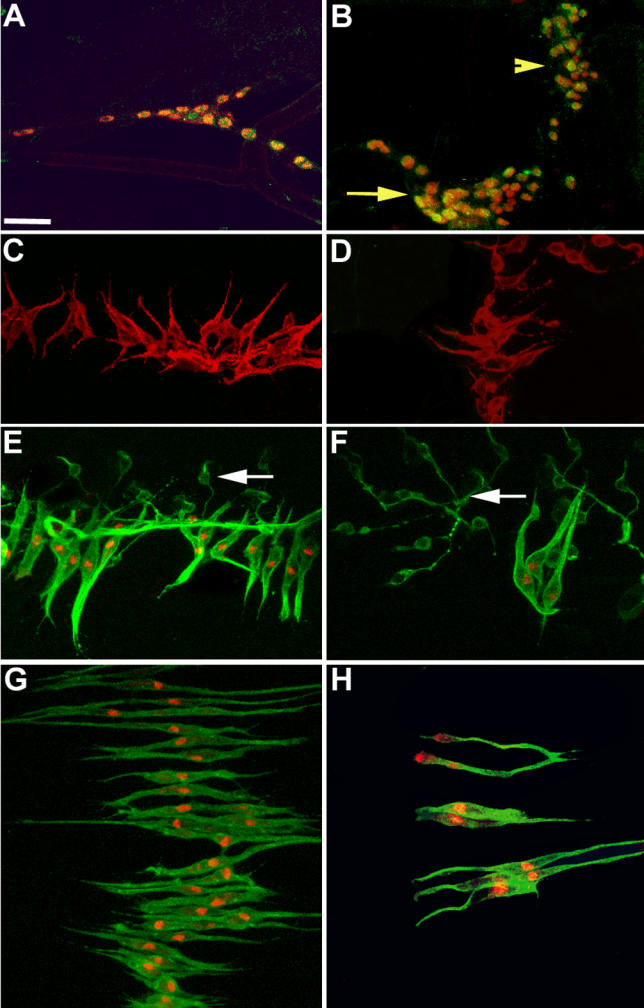
sty and Founder Cells (A and B) *sty-lacZ* third instar larva (A) and pupa 20 h APF (B) double-labelled with anti-Twi (red) and anti-β-galactosidase (green). (A) A dorsal cluster of Twi-expressing myoblasts, all expressing *sty-lacZ*. (B) A dorsal cluster (yellow arrow) and a lateral cluster (yellow arrowhead) of myoblasts in a 20 h APF pupa. Not all myoblasts express *sty-lacZ* at this stage. (C and D) *sty-lacZ* pupa 28 h APF double-labelled with 22C10 (red) and anti-β-galactosidase (green). *sty-lacZ* is not expressed in the dorsal (C) or lateral (D) set of founders. (E–H) Pupae 28 h APF (grown in 29 °C) double-labelled with 22C10 (green) and anti-β-galactosidase (red). (E and G) *duf-lacZ* pupae showing sets of dorsal (E) and lateral (G) founders. (F and H) *1151/duf-lacZ; UAS-sty/+* pupae showing decreased number of founders in the dorsal (F) and lateral (H) hemi-segments. White arrows in (E) and (F) indicate 22C10-labelled neuronal branching. Anterior is at top; dorsal midline is at left. Scale = 30 μm.

Both Htl and Hbr/Dof are expressed in undifferentiated myoblasts prior to the founder cell selection stage. However these myoblasts do not up-regulate *duf-lacZ* expression—the characteristic property of adult founders—showing that Htl, if active, does not specify founders at this stage. This could be because the ligand is not available or because of the presence of negative regulators. Dof-mediated mis-expression resulted in an increased founder and fibre number phenotype. Since Hbr/Dof function is ligand-dependent, this implies that the ligand, like the receptor, is generally available. Thus, there must be another factor that prevents premature receptor activation during normal development. We tested whether this factor might be encoded by *sty*. Sty is a negative regulator of FGF signalling [49–51]. We examined *sty-lacZ* expression in adult myoblasts. In the third larval instar, *sty-lacZ* expression was seen in all nerve-associated adult myoblasts (Figure 7). This expression declined gradually in the pupal stages. Figure 7B shows that *sty-lacZ* expression occurs only in a subset of cells at 18 h APF. By 28 h APF, when founders are chosen in the abdomen, *sty-lacZ* expression was not detected in adult myoblasts (Figure 7C and 7D). We do not know how exactly the expression of *sty-lacZ* correlates with that of Sty. However, these results are suggestive of a negative regulatory role of Sty that prevents premature receptor activation.

### Over-Expression of *sty* in Myoblasts Leads to Absence of Founder Cells

We maintained expression of *sty* in myoblasts by expressing *UAS-sty* under the *1151-GAL4* driver. *sty* mis-expression led to a discernible decrease in the number of founder cells. In contrast to wild-type numbers of about 17–22 dorsal founders and about 20 lateral founders per hemi-segment, *1151*/*duf-lacZ; UAS-sty/+* pupae had an average of seven dorsal founders and eight lateral founders per hemi-segment (*n =* 15). Figure 7F and 7H show, respectively, the decreased number of dorsal and lateral founders in *1151/UAS-sty* pupae in comparison to the controls (Figure 7E and 7G). This suggested that down-regulation of *sty* is a prerequisite for cells to adopt a founder fate. We examined the status of the Twi-expressing myoblasts in *1151*/+; *UAS-sty/+* pupae at 28 h APF (Figure S1D). Sty over-expression produced no comparable change in the number of fusion-competent myoblasts when compared to the control (dorsal myoblasts, wild-type, 96 ± 8, *UAS-sty,* 84 ± 5, *n =* 4; qualitatively similar results obtained for lateral myoblasts). This result, along with the results of *sty-lacZ* expression and *sty* mis-expression*,* support a direct role of *sty* in founder cell selection.

## Discussion

During somatic myogenesis in the *Drosophila* embryo, combinatorial functions of the Wingless, Decapentaplegic, and Ras pathways determine domains of mesodermal cells in each segment, from which a single precursor cell is chosen by Notch-mediated lateral inhibition [1,2,21]. The daughters of the precursor cell form two embryonic muscle founder cells—each with a characteristic pattern of expression of markers that specify its identity—or they form an embryonic muscle founder cell and an adult myoblast progenitor [1]. This latter cell type proliferates during larval life and its progeny, the adult myoblasts, are associated with imaginal discs and larval nerves. While embryonic founder cells shut down the expression of Twi, a marker of myoblast identity, the adult myoblasts retain Twi expression during their proliferative phase during larval life [52]. At the onset of metamorphosis, Twi levels decline in a group of cells, the adult founders, that express *duf-lacZ* at high levels and are located at the sites of myofibre formation [15]. Twi expression is also shut off in other myoblasts as they fuse with the founder to form multi-nucleate cells [53].

Interestingly, adult myoblasts, like the embryonic founders from whose siblings they are derived, express *duf-lacZ* (albeit at low levels) throughout larval life [15]. As adult muscle differentiation begins, this low-level expression changes dramatically to a pattern in which one founder cell—expressing *duf-lacZ* at high levels—is chosen to seed each muscle fibre [15]. How is this founder cell chosen? We have shown earlier that removal of Notch signalling in adult myoblasts does not result in an increase in the number of founders [15]. This suggested that lateral inhibition mediated by Notch, the process that operates in the embryo, is not the mechanism by which adult founders are chosen. Indeed, the requirements are quite different, for adult myoblasts all express *duf-lacZ* at low levels, suggesting—consistent with their origins as siblings of embryonic founders—that they all already have some properties similar to founder cells. In choosing adult founder cells, therefore, *duf-lacZ* is to be up-regulated in cells that will become founders and down-regulated in others that will become fusion-competent cells. Our results show that the Htl pathway plays a key role in choosing adult founders. We also suggest that Htl does this using an unusual mechanism in which an intracellular positive regulator plays an important role.

Adult myoblasts in the third larval instar express Twi, Hbr/Dof, Htl, and *sty-lacZ*. At the onset of adult abdominal myogenesis, Twi expression declines. With this, the expression of Hbr and Sty declines in myoblasts. We suggest that, in the third instar larva, the presence of Sty prevents the activation of the Htl receptor, even if the ligand and Hbr/Dof are available. However, since both Hbr/Dof and *sty-lacZ* expression decline with Twi, at the onset of myogenesis, the Htl receptor will still be unable to function, because Hbr/Dof is necessary for the function of the Htl receptor. We would like to suggest that, as Sty and Hbr/Dof expression decline (as Twi expression shuts down at the onset of myogenesis), the Htl receptor is active in some myoblasts. Htl signalling maintains Hbr/Dof expression in these cells by a positive feedback mechanism. Maintenance of Hbr/Dof expression reinforces the Htl signal, which in turn up-regulates the expression of founder-specific genes such as *duf* in these cells, thereby imparting them with founder properties. Consistent with this hypothesis, activating the Htl receptor results in the maintenance of Hbr/Dof in adult myoblasts. This prolonged activation of Hbr/Dof, and therefore of *duf*, could be the cause of morphological changes associated with the excess founder cells, as observed in Figures 4B and 6B.

How could this localised activation of the receptor occur? One way is via the localised availability of the Htl ligand. Proximity of some of the cells to the source of the ligand could cause higher levels of Htl signalling in those cells than others, thus biasing their fate towards that of a founder. Examining the expression pattern of the recently identified ligands of Htl [54,55] should be able to resolve whether this indeed is the case. A second, and more likely, mechanism for localised activation of receptor is via a process that does not involve the localised presence of the ligand. We suggest this possibility because the continued mis-expression of Hbr/Dof in all adult myoblasts results in an increased number of founders and muscle fibres. Since Hbr/Dof function is dependent on ligand activation of the receptor, the ligand must be available to Htl on all myoblasts. Local activation of the receptor could occur by Hbr/Dof being maintained briefly in a founder cell pattern in some myoblasts even as all of the others down-regulate Sty and Hbr/Dof at the onset of myogenesis (with the decline of Twi expression). This continued expression of Hbr/Dof in some myoblasts, and the absence of Sty, could allow local activation of the receptor and the consequent maintenance of Hbr/Dof in a founder pattern.

The problem then shifts to deciphering the mechanism by which the (hypothetical) localised activation of Hbr/Dof takes place. Since abdominal myoblasts are associated with nerves, one possibility is that the signal could come from the nerves. This “solution” has two problems, however. First, it is not clear how a precise periodicity of signal, expressed along the nerve and seen by associated myoblasts, would be generated to organise the correct spacing of founder cells. More pertinent perhaps is the observation that surgical removal of the nerve does not affect the number of muscle fibres [56]. Thus, nerves are unlikely to be the source for the signal that organises myoblasts in a founder pattern. Another possible source for a signal that maintains and elevates Hbr/Dof expression in a founder pattern could be the epidermis. The abdominal epidermis develops from ectodermal cells, the histoblasts [57]. As the epidermis differentiates during metamorphosis, muscle tendon precursor cells—specified by and expressing the *stripe* locus—can be identified [58]. The tendon precursor cells, given that they are in proximity to the differentiating myoblasts, could possibly be a source of organising signal that modulates Hbr/Dof expression to a founder pattern. Thus, the precise segmental and regional patterning of the epidermis could organise the pattern of founder cells in the developing abdominal musculature. In favour of this hypothesis is the finding that reduction of *stripe*-expressing cells in the dorsal thoracic disc results in the reduction of *duf-lacZ* expression in the larval templates that give rise to the thoracic dorsal longitudinal muscles [53], and increasing *stripe* expression in the ectoderm results in the increase of *duf-lacZ* expression in the developing dorsal longitudinal muscles (Arjumand Ghazi and K. VijayRaghavan, unpublished data). We do not know yet if these results apply to the abdomen.

A third possible mechanism of localised activation of Htl, not exclusive of either of the ones mentioned earlier, is that a dynamic interaction between ligands, other activators, and repressors results in the activation of Htl in a specific pattern. Such a process has been described in the embryo, e.g., in the anterior patterning of follicle cells in the *Drosophila* egg [59].

In conclusion, while many mechanistic details still remain elusive, the implication of the FGF pathway as a key player in adult founder cell choice provides the molecular tools to identify missing elements in the pathway. Integrated within the broad question of founder cell specification are more specific questions pertinent to the different muscle groups. Activation of Htl signalling produces a less prominent effect on the dorsal muscles than on the lateral muscles. Also, the extra founders of the dorsal muscles are located in a characteristic fashion (altered in orientation; see Figure 4H) that is different from that observed for the excess lateral founders. These observations raise questions about whether the dorsal and lateral groups of founders have different levels of sensitivity to the FGF pathway and whether they employ the pathway in different ways.

Our results allow the testing of whether this pathway operates in a similar manner during myogenesis in other contexts in *Drosophila* and in other animals, in particular the higher vertebrates. Vertebrate muscles are composed of multiple fibres, which make them similar to *Drosophila* adult muscles [15,60]. Vertebrate myogenesis shares several features with *Drosophila* myogenesis, at the level of genetic and molecular regulatory mechanisms [28,61]. The FGF pathway in vertebrates, mediated by multiple isoforms of the receptor and the ligand, has been found to play an instructive role in induction and commitment of myogenic cells. In *Xenopus,* for instance, an FGF-mediated pathway controls specification and differentiation of myotomal progenitors [62]. Also, signalling via FGFR4 positively regulates myogenic differentiation during avian limb muscle development [63]. The present study, showing the role of Htl in muscle differentiation, highlights yet another similarity. Our study also provides directions for probing how the number and location of fibres are regulated in vertebrates, questions that remain to be resolved in the field of vertebrate myogenesis.

## Materials and Methods

### Fly strains

To follow *duf* expression, the enhancer-trap line rp298 [14], which has a P element nuclear-localising lacZ inserted within the promoter region of *duf,* was used [11]. The GAL4-UAS system [64] was used for directed expression of genes during adult myogenesis. The *1151-GAL4* enhancer-trap strain, obtained from L. S. Shashidhara (Centre for Cellular and Molecular Biology, Hyderabad, India), is expressed in all adult myoblasts associated with the imaginal discs and nerves in the larvae [27,65]. *UAS-htl-RNAi* was obtained from Arno Müller (Institute für Genetik, University of Dusseldorf, Germany). *UAS-yan^ac^* ^t^, *UAS-sty,* and *sty-lacZ* were gifts from Ben-Zion Shilo (Weizmann Institute of Science, Rehovot, Israel). *UAS-dof* was a gift from Maria Leptin (Institute für Genetik, University of Cologne, Germany). *UAS-GFPN-lacZ/Cyo; btl-GAL4/TM3, Sb Ser* was a gift from Shigeo Hayashi (Riken Center for Developmental Biology, Kobe, Japan). The following stocks were obtained from the Bloomington *Drosphila* Stock Center (http://flystocks.bio.indiana.edu/: *UAS-dnDER*, *UAS-aos*, *UAS-dnhtl*, *UAS–activated htl* (or *UAS-*λ*htl*), *UAS-pntP1*, and *tub-GAL80ts*. The various UAS strains (except the strains *UAS-dnhtl, UAS-dnDER,* and *UAS-aos,* which have double copy of the UAS insert) were crossed into the background of *duf-lacZ* using standard genetic techniques. For characterising the expression profile of genes and proteins, fly stocks and crosses were grown at 25 °C. For the GAL4-UAS mis-expression studies, progeny of the crosses and their controls were grown at 25 °C until early second instar stages, and then shifted to 29 °C until the required pupal stages.

### Tissue preparation

Wandering third instar larvae were collected for larval dissections. White pre-pupae (0 h APF) were collected and grown at appropriate temperatures for desired times prior to dissection. The pupal and larval tissues were prepared for immunohistochemistry as described previously [53]. The larval or pupal preparations were mounted in 70% glycerol for DAB (di-amino benzidine)–stained preparations, or in Vectashield mounting medium (Vector Labs, Burlingame, California, United States) for fluorescent-labelled preparations.

### Immunohistochemistry

Mouse anti-β-galactosidase antibody and 22C10 (both from Developmental Studies Hybridoma Bank; http://www.uiowa.edu/~dshbwww/) were used at a dilution of 1:50. Rabbit anti-β-galactosidase antibody (obtained from Molecular Probes, Eugene, Oregon, United States) was used at a dilution of 1:5,000. Rabbit anti-Twi antibody, a gift from Siegfried Roth (University of Cologne), and Rabbit anti–Myosin heavy chain (MHC) antibody, a gift from Dan Kiehart (Duke University, Durham, North Carolina, United States), were used at a dilution of 1:500. Rabbit anti-Dof antibody, a gift from Maria Leptin (Institute für Genetik, University of Cologne), was used at a dilution of 1:200. Rabbit anti-Htl antibody, a gift from Alan Michelson (Brigham and Women's Hospital, Boston, Massachusetts, United States), was pre-adsorbed at a dilution of 1:2,000 and used. For the anti-Htl antibody, the signal was amplified by Tyramide amplification kit (TSA, NEN Life Science Products, Boston, Massachusetts, United States). Tyramide was diluted in the amplification solution at a dilution of 1:50, and the streptavidin-conjugated Alexa 568 was used at a dilution of 1:200. Secondary antibodies conjugated to Alexa Fluor dyes (Molecular Probes) were used at a dilution of 1:200—Alexa 488 for green and Alexa 568 for red labelling.

### Microscopy and cell count

Fluorescent preparations were scanned using a confocal microscope (MRC-1024, Bio-Rad Laboratories, Hercules, California, United States), and images were analysed using the software Metamorph (version 4.5) (Molecular Devices, Sunnyvale, California, United States). The DAB-stained preparations were examined using a Nikon (Tokyo, Japan) Eclipse E1000 microscope. For myoblast counts in wild-type and experimental pupae, abdominal segments A2 to A6 were considered.

## Supporting Information

Figure S1 Status of Twi-Expressing Myoblasts upon Modification in Levels of Htl Signalling Fluorescent preparations of pupae, grown for 28 h at 29 °C, stained with anti-Twi antibody to label the myoblasts. A cluster of dorsal myoblasts is in view in each of the images. See text for supporting quantitative data for each experiment.
(A) Wild-type pupa.
(B) *1151/+*; *UAS-dnhtl/+*; *UAS-dnhtl/+* pupa. The number of myoblasts remains unaffected.
(C) *1151/+*; *UAS-*λ*htl/+* pupa. A moderate increase in myoblast numbers is observed in this case.
(D) *1151/+*; *UAS-Sty/+* pupa. The number of myoblasts remains largely unaffected.
(3.9 MB TIF).

Figure S2 Founder Number and Myoblast Number upon Modification of Htl Levels Using the *tub-GAL80ts* System Fluorescent images of pupae stained with antibodies 22C10 (red), to mark the founders, and anti-Twi (green), to mark the myoblasts. *tub-GAL80ts* [44] strain was put in the background of *1151-GAL4* using standard genetic techniques. The temperature-sensitive GAL80 protein is functional at 18 °C and represses the activating function of GAL4 protein. At a higher temperature (29 °C), the GAL80ts becomes non-functional and the GAL4 can activate the genes downstream of *UAS* sequence [44]. *1151-GAL4; tub-GAL80ts* stock was crossed, separately, to *UAS-*λ*htl* and *UAS-dnhtl* stocks. The progeny of the above crosses were grown for 30 h at 18 °C, followed by 11 h at 29 °C. This timing corresponds to a stage prior to founder selection in wild-type. Pupae of *1151-GAL4; tub-GAL80ts* were also treated similarly, to serve as control. To check whether the GAL80ts protein was functional in the pupal mesoderm in particular, *1151-GAL4, UAS-*λ*htl, tub-GAL80ts* pupae were grown for 50 h at 18 °C followed by 4 h at 29 °C.
(A) *1151-GAL4/+*; *tub-GAL80ts/+* pupa grown for 30 h at 18 °C followed by 11 h at 29 °C (control). A set of lateral founders (one of them indicated by white arrow) is in view. The Twi-expressing myoblasts are seen aligned over the founders.
(B) *1151-GAL4; tub-GAL80ts; UAS-*λ*htl* pupa grown for 50 h at 18 °C followed by 4 h at 29 °C. At 18 °C, the GAL80 protein is functional and represses GAL4 activation of *UAS-*λ*htl*. The founders and Twi-expressing fusion-competent myoblasts are present in wild-type pattern.
(C) *1151-GAL4; tub-GAL80ts; UAS-*λ*htl* pupa grown for 30 h at 18 °C, followed by 11 h at 29 °C (i.e., similarly treated as in [A]). Founders are present in clusters (indicated by white arrows). The fusion-competent myoblasts are not aligned in a pattern similar to that observed in (A) or (B), but their number does not change significantly (see text).
(D) Dorsal region of an *1151-GAL4; tub-GAL80ts; UAS-dnhtl* pupa similarly treated as in (A). Twi-expressing cells are present (white arrowhead) but founders are not observed.
For (A–C), anterior is at left; dorsal midline is at top. For (D), anterior is at top; dorsal midline is at left.
(2.5 MB TIF).

## Abbreviations

Aos: Argos
*APF*: after puparium formation
*btl*: *breathless*
*duf*: *dumbfounded*
DER: *Drosophila* Epidermal growth factor receptor
dnHtl: dominant-negative construct of Heartless
Dof: Downstream of fibroblast growth factor receptor
FGF: Fibroblast growth factor
FGFR: Fibroblast growth factor receptor
GAL80ts: temperature-sensitive GAL80
Hbr: Heartbroken
Htl: Heartless
MAPK: mitogen-activated protein kinase
MHC: Myosin heavy chain
Pnt: Pointed
RTK: receptor tyrosine kinase
*tub*: *tubulin*
*Sty*: Sprout
Twi: Twist
*UAS-dnDER*: dominant-negative construct of *Drosophila* Epidermal growth factor receptor
Yan^act^: activated Yan
